# Acoustic Anomalies and Phase Transition Behaviors of Lead-Free Piezoelectric (Na_1/2_Bi_1/2_)TiO_3_-*x*BaTiO_3_ Single Crystals as Revealed by Brillouin Light Scattering

**DOI:** 10.3390/ma11061000

**Published:** 2018-06-12

**Authors:** Byoung Wan Lee, Soo Han Oh, Jae-Hyeon Ko, Xiaobing Li, Haosu Luo

**Affiliations:** 1 Department of Physics, Hallym University, Chuncheon, Gangwon-do 24252, Korea; simponi12@gmail.com (B.W.L.); soohanoh@naver.com (S.H.O.); 2 Shanghai Institute of Ceramics, Chinese Academy of Sciences, Shanghai 201800, China; lxbing@mail.sic.ac.cn (X.L.); hsluo@mail.sic.ac.cn (H.L.)

**Keywords:** NBT-BT, lead-free, relaxor, polar nanoregion, Brillouin scattering

## Abstract

The elastic properties of unpoled and prepoled (Na_1/2_Bi_1/2_)TiO_3_-*x*BaTiO_3_ (NBT-*x*BT) single crystals near the morphotropic phase boundary were investigated as a function of temperature using Brillouin light scattering. The acoustic mode frequency and the related acoustic damping of unpoled NBT-*x*BT showed very broad minimum and maximum, respectively, consistent with typical relaxor behaviors. The frequency softening of the longitudinal acoustic mode together with the increase in acoustic damping was largest along the <100> direction, indicating that polarization fluctuations were most substantial along this crystallographic direction. The difference in acoustic behaviors between the unpoled NBT-*x*BTs with *x* = 0.05 and 0.08 were negligible, which means that the NBT-*x*BT system exhibits typical relaxor properties over a certain composition range of at least 5~8%. The obtained relaxation time of polar nanoregions in the paraelectric phase showed a gradual slowing-down character without any critical divergent behavior. The prepoling of NBT-*x*BT along the <100> direction induced drastic changes in both mode frequency and damping at ~110 °C when the poling field was larger than 1.4 kV/mm, corresponding to the depoling process from macroscopic/mesoscopic ferroelectric order to ergodic relaxor state upon heating. Phase coexistence of ferroelectric and relaxor states was observed at the intermediate poling field of 1.4 kV/mm.

## 1. Introduction

Piezoelectric materials are indispensable to numerous devices that convert mechanical energy into electric energy and vice versa [1,2]. Recent efforts in the development of lead-free piezoelectric materials have opened a new possibility for replacing Pb-based materials with environment-friendly ones, although the piezoelectric performances of Pb-free materials are generally inferior to those of Pb-based systems [3]. (1 − *x*)(Na_1/2_Bi_1/2_)TiO_3_-*x*BaTiO_3_ (NBT-*x*BT) is an archetypal lead-free material showing relatively good piezoelectric performance at compositions near the morphotropic phase boundary (MPB) [4,5,6]. The MPB composition of NBT-*x*BT, which divides the (Na_1/2_Bi_1/2_)-rich rhombohedral phase and the Ba-rich tetragonal phase, is approximately 6~7%, at which the piezoelectric properties become maxima. It was reported that poled NBT-*x*BT near the MPB showed an electromechanical coupling coefficient of 62% and a piezoelectric coefficient *d*_33_ of 360 pC/N along the <100> direction [5]. Many studies have focused on the microscopic nature of MPB compositions [7,8,9]. For example, the possibility of tuning of MPB, i.e., the creation and destruction of MPB by electrical poling, has been suggested [10]. 

Unpoled NBT-*x*BT near the MPB exhibits typical relaxor behaviors, such as broad dielectric permittivity peaks together with significant frequency dispersion and the formation of nanoscale local polar regions without any macroscopic long-range order [6,11]. The average symmetry of the unpoled NBT-*x*BT near the MPB is cubic, while TEM (transmission electron microscopy) and ^23^Na NMR (Nuclear Magnetic Resonance) measurements show that the cubic phase coexists with other local polar distortions of lower symmetry [6,12]. Another TEM study showed that the volume fraction of tetragonal polar nanoregions (PNRs) increases as the BT content increases toward the MPB [9]. The relation between the local structure and the relaxor behavior, as well as the relation between the local structure and the average structure, has been considered as an important subject and investigated intensively [13,14,15]. A more recent NMR study has suggested that the octahedral tilting disorder is responsible for the relaxor behaviors of NBT-*x*BT [16]. 

Another interesting aspect of the NBT-*x*BT system is the effect of electric fields on phase and structural changes. Evolution of irreversible structural transformation and formation of long-range polar order under an electric field have been reported for NBT-*x*BT near the MPB compositions in detail [17,18]. In addition, it has been shown that Mn-doping enhances the ferroelectric and piezoelectric properties of NBT-*x*BT [19]. Successful growth of high-quality NBT-*x*BT single crystals has opened a new possibility of investigating fundamental properties, such as elastic and optical properties [5,19,20]. Vibrational properties of the optical phonons of NBT-*x*BT have been investigated in terms of Raman spectroscopy [21,22,23], but studies on the low-frequency acoustic phonon modes are rather scarce [24,25,26,27]. The acoustic properties are related to elastic waves propagating in the sample and can be used to determine elastic constants, which are a fundamental physical property. Cordero has shown the important relationship between elastic properties and enhanced piezoelectric performance of several ferroelectric materials at the MPB [28]. 

One of the experimental methods to investigate elastic properties is Brillouin spectroscopy, which probes the frequency and attenuation (damping) of acoustic waves in the gigahertz range [29]. The strains caused by high-frequency acoustic waves can be coupled to fast dynamics in condensed matters. Evolution of polar structures as a function of temperature has been an important subject in the field of ferroelectric relaxors [30]. The concept of PNRs is considered to play an important role in the formation of the complex structural and dynamic properties of relaxors. The fast dynamics of PNRs can be investigated in terms of Brillouin scattering in the high-temperature phase. The purpose of this study is to probe the elastic properties of NBT-*x*BT with *x* = 5% (and, to a lesser extent, 8%) near the MPB along different crystallographic directions, and to discuss the coupling between acoustic waves and the dynamics of PNRs. 

## 2. Results

### 2.1. Brillouin Scattering Spectrum

**Figure 1:**
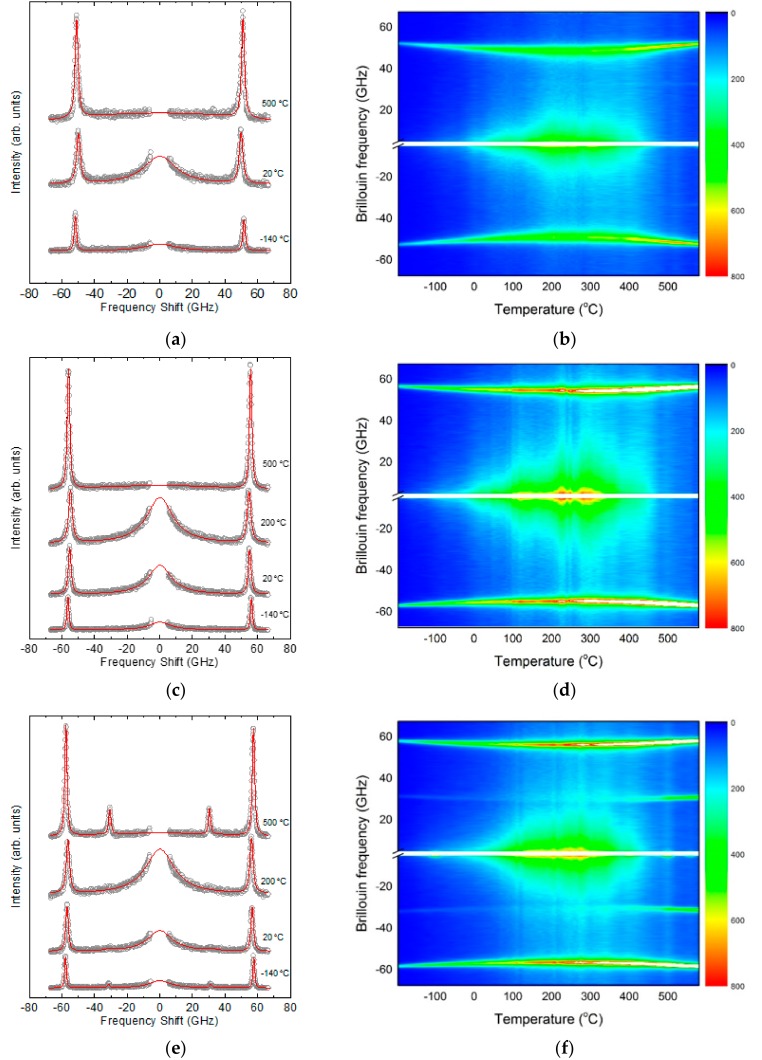
Temperature dependence of the Brillouin scattering spectra of NBT-5%BT for the phonon propagation direction along the (**a**) <100>, (**c**) <110>, and (**e**) <111> directions. The solid lines denote the best-fitted results as described in the text. The right figures (**b**), (**d**), (**f**) show the intensity color plots for the Brillouin spectra of each corresponding direction.

Figure 1a,c,e shows temperature dependences of the Brillouin spectrum measured along the crystallographic <100>, <110>, and <111> directions, respectively. All spectra exhibit strong resonance peaks at around 50–60 GHz, which corresponds to the longitudinal acoustic (LA) phonon mode propagating along each direction. On the other hand, the spectra measured along the <111> direction show additional peaks at ~31 GHz. This low-frequency mode for the <111> direction corresponds to the transverse acoustic (TA) mode of NBT-5%BT. Figure 1b,d,f shows the intensity color plots for the Brillouin spectra measured along the <100>, <110>, and <111> directions, respectively. The central white line denotes the Rayleigh line corresponding to excitation laser frequency. The trace of the LA mode is clearly seen from the three-color plots, while a weak trace of the TA mode is seen only from the data measured along the <111> direction. In addition, wing-like features can be observed around the Rayleigh line at temperatures between approximately 400 and 0 °C. This represents quasielastic central peaks (CP) representing relaxational dynamics.

### 2.2. Acoustic Mode Behaviors

**Figure 2:**
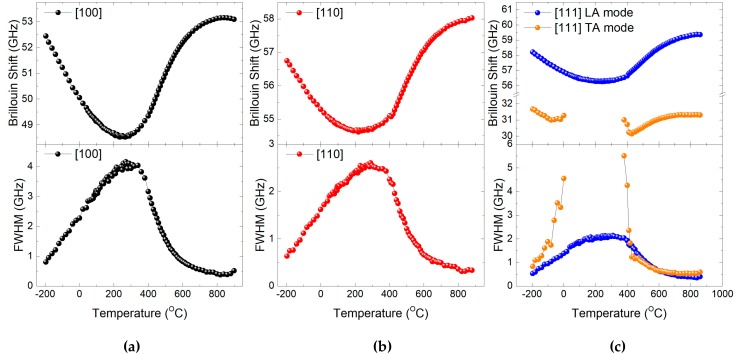
Temperature dependences of Brillouin frequency shift (*ν*_B_) (upper figures) and full width at half maximum (FWHM, Γ_B_) (lower figures) of the longitudinal acoustic (LA) and transverse acoustic (TA) modes for the (**a**) <100>, (**b**) <110>, and (**c**) <111> directions.

The response function of the damped harmonic oscillator, which is approximated by the Lorentzian function, was used to fit the Brillouin spectrum. The instrumental Gaussian function of the interferometer was considered during the curve-fitting. The solid lines in Figure 1a,c,e are the best-fitted lines, from which the Brillouin frequency shift (*ν*_B_) and the full width at half maximum (FWHM, Γ_B_) of the acoustic modes could be obtained as a function of temperature. The *ν*_B_ is linearly proportional to the sound velocity of the acoustic modes, while the Γ_B_ represents the attenuation coefficient of the acoustic waves representing their damping behavior. Figure 2a–c shows the temperature dependences of *ν*_B_ and Γ_B_ for the <100>, <110>, and <111> directions, respectively. Because there is no reported information about the temperature dependence of the refractive index, sound velocities could not be calculated from the present data.

All the LA modes show softening in their mode frequencies along the three crystallographic directions. The onset of softening was located at high temperatures around 800 °C, which is much higher than those of typical relaxors such as Pb(Mg_1/3_Nb_2/3_)O_3_ (PMN) and Pb(Mg_1/3_Ta_2/3_)O_3_ (PMT) [31,32,33]. Acoustic mode softening usually occurs due to the coupling of acoustic waves and other degrees of freedom. Since there is no macroscopic polarization in the unpoled NBT-5%BT system, the most probable degree of freedom that couples to the acoustic waves is the PNRs. The polarization fluctuation damping of PNRs is the main origin of the large acoustic attenuation represented by the broad damping peak of FWHM. The degree of softening, i.e., the change in mode frequency, is largest along the <100> direction, which is accompanied by the highest Γ_B_ along the same direction. The degree of softening of the LA mode frequency *ν*_B_, which is defined by the change in the mode frequency between the maximum and the minimum values, is 4.56, 3.41, and 3.08 GHz for the <100>, <110>, and <111> directions, respectively. Similarly, the growth of the Γ_B_, estimated from the difference between the maximum at ~250 °C and the background value at high temperatures above 800 °C, is 3.76, 2.27, and 1.73 GHz, respectively. Since the mode softening of acoustic waves usually begins to occur near the Burns temperature in typical relaxors [31,32,33], we may consider the onset temperature of ~800 °C as the approximate Burns temperature of NBT-5%BT. 

It is interesting to note that the TA mode propagating along the <111> direction is heavily damped in a temperature range of 20~360 °C, thus, the TA mode is hardly seen in this range. This suggests that the TA mode couples to some dynamic degrees of freedom in the system, which we presume is the PNRs. This coupling dissipates the energy of TA waves very severely in a certain temperature range. It is interesting to note that the softening of the TA mode seems to occur at a lower temperature of ~700 °C. A similar phenomenon was observed in typical relaxors such as PMN [32]. 

### 2.3. Quasielastic Central Peaks

The acoustic mode behaviors shown in Figure 2 suggest that the unpoled NBT-*x*BT system near the MPB exhibits typical relaxor behaviors. The broad minimum of *ν*_B_ and the concomitant broad maximum of Γ_B_ without any sharp, discontinuous changes indicate that there is no long-range ferroelectric order at low temperatures. This is consistent with the recent ^23^Na NMR results, which showed that NBT-*x*BTs near the MPB exhibit substantial octahedral tilting disorder and relaxor states [16]. The microscopic origin of the gradual mode softening of the LA mode is attributed to the coupling between the strains caused by acoustic waves and local polarizations of the dynamic PNRs [34]. The relaxational motions of PNRs induce quasielastic central peaks (CPs) near the Rayleigh line [35]. Figure 1b,d,f clearly shows that CPs appear in a specific temperature range of 0~450 °C, exactly the same range in which the minimum of *ν*_B_ and the maximum of Γ_B_ appear. This coincidence suggests that the relaxation process causing the CPs is also responsible for the acoustic anomalies of the LA and TA modes.

### 2.4. Effect of BT Composition on Acoustic Anomalies

**Figure 3:**
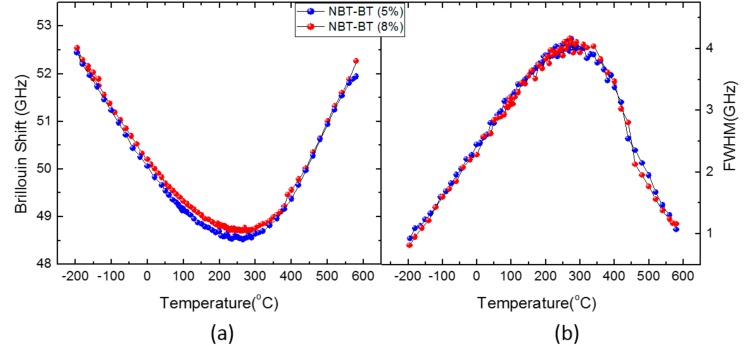
Temperature dependences of (**a**) *ν*_B_ and (**b**) Γ_B_ of the LA mode of the two unpoled (Na_1/2_Bi_1/2_)TiO_3_-*x*BaTiO_3_ (NBT-*x*BT) with *x* = 5% and 8% single crystals. The phonon propagation direction is along the <100> direction.

Figure 3a,b shows the temperature dependences of *ν*_B_ and Γ_B_, respectively, of the LA mode propagating along the <100> direction in NBT-5%BT and NBT-8%BT single crystals. Figure 3 shows that the broad acoustic anomalies of NBT-*x*BT do not change appreciably, at least in the BT concentration of 5~8%. The LA mode of the two crystals exhibits surprisingly the same behaviors over the whole temperature range below 600 °C. This indicates that, without any prepoling, NBT-*x*BT shows typical relaxor behaviors in the BT composition of 5~8% near the MPB. This result is consistent with a recent dielectric study, which determined that relaxor state is maintained over a specific composition range near the MPB [16]. 

### 2.5. Effect of Electric Field on Acoustic Anomalies

**Figure 4:**
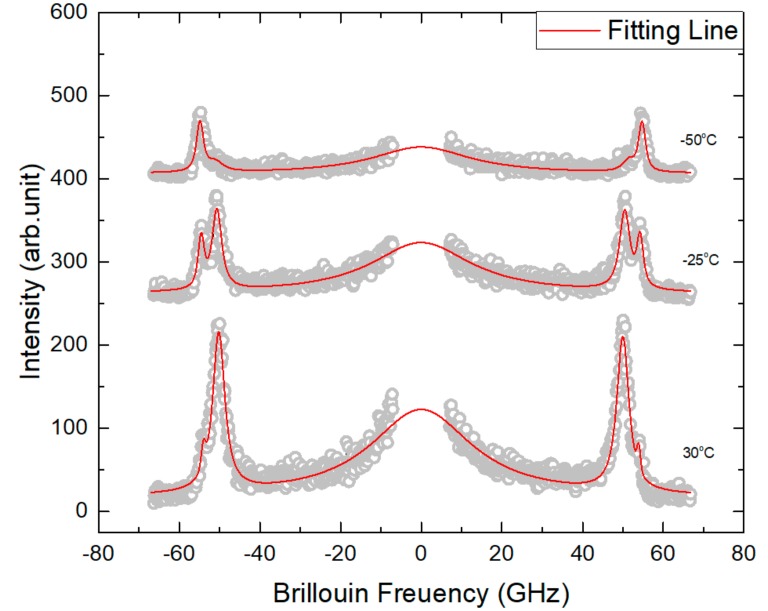
Temperature dependence of the Brillouin spectrum of NBT-*x*BT with *x* = 5% prepoled at room temperature under 1.4 kV/mm. The phonon propagation direction is along the <100> direction.

**Figure 5:**
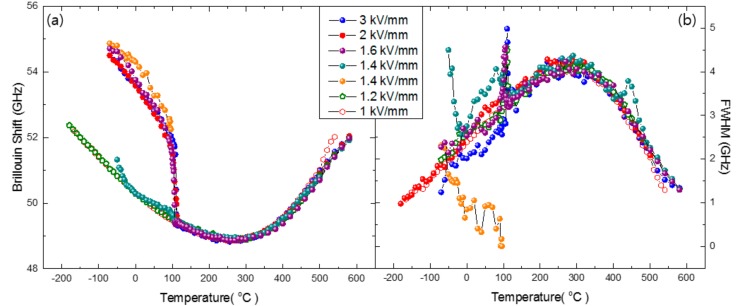
Temperature dependence of (**a**) *ν*_B_ and (**b**) Γ_B_ of the LA mode of NBT-*x*BT with *x* = 5% prepoled at room temperature under several electric fields. The phonon propagation direction is along the <100> direction.

Figure 4 shows the temperature dependence of the Brillouin spectrum of NBT-5%BT prepoled at room temperature under the electric field of 1.4 kV/mm applied along the [100] direction. The sample was prepoled at room temperature, cooled to the lowest temperature of −196 °C, and the Brillouin spectrum was then measured upon heating. The LA mode clearly shows splitting in a certain temperature range. Two Lorentzian functions were used to obtain the *ν*_B_ and Γ_B_ of each LA mode component. However, because of the proximity of the two components, the fitting quality was not good at some temperatures, which is the reason for the rather scattered values of the half widths shown in Figure 5b. 

Once the electric field becomes larger than 1.4 kV/mm, the LA mode does not exhibit any splitting. Figure 5 shows the dependence of the Brillouin shift and the FWHM of NBT-5%BT prepoled under different electric fields. If the electric field is smaller than 1.4 kV/mm, the acoustic properties of the prepoled NBT-5%BT are identical to those of the unpoled sample. On the other hand, the LA mode exhibits clear splitting once the electric field reaches 1.4 kV/mm. The splitting disappears at ~110 °C, above which there is no difference in acoustic properties between poled and unpoled NBT-5%BT samples. This temperature corresponds to the depolarization temperature *T_d_* of NBT-*x*BT near MPB. Among the two components of the split LA mode prepoled at 1.4 kV/mm, the low-frequency component of the LA mode represents relaxor behavior since it is the same as the mode frequency of the unpoled NBT-5%BT. On the other hand, the high-frequency component is associated with the mesoscopic or long-range ferroelectric order caused by external electric field. The sharp acoustic damping at ~110 °C represents the transformation of the mesoscopic ferroelectric order into PNRs. In this context, the splitting of the LA mode at low temperatures suggests the coexistence of relaxor state and mesoscopic/long-range ferroelectric order. A further interesting result is that the FWHM exhibits a very sharp peak at ~110 °C, at which the Brillouin shift shows a precipitous change. Similar damping peaks have been observed in Pb(Mg_1/3_Nb_2/3_)O_3_-PbTiO_3_ (PMN-PT) complex perovskite relaxors near the MPB composition [33]. 

## 3. Discussion

The softening of the LA mode and the substantial increase in the FWHM are caused by the electrostrictive coupling between the PNRs and acoustic waves. It is known that PNRs are formed at a certain temperature, known as Burns temperature (*T_B_*). *T_B_* of PMN is 620 K while that of NBT-5%BT is presumed to be approximately 1100 K, which is the onset temperature of the mode softening [27]. Spectroscopic study revealed local ordering of Na-Bi clusters [36], suggesting it to be one of the origins of PNRs in this system. Recent neutron and x-ray scattering measurements showed an extensive diffuse scattering network, indicating a short-range ordering of polar regions [14]. The relaxation process of the PNRs interacts with the strain perturbation caused by the propagating acoustic waves, generally resulting in a decrease in the effective elastic constant *C*. The anomalous changes in the real and imaginary part of the elastic constant, denoted as Re[Δ*C*] and Im[Δ*C*], respectively, are expressed in terms of the following equations based on the phenomenological Slonczewski–Thomas approach [34]:

(1) Re[ΔC]=−γ2Plocal2ε1+ω2τ2∝Plocal2,

(2) Im[ΔC]=(ωτ)γ2Plocal2ε1+ω2τ2∝Plocal2.

In these equations, *γ* is the electrostrictive coefficient between the strain *ε* and the squared local polarization *P_local_*. *ω* is the angular frequency of the acoustic waves and *τ* the relaxation time of the relevant relaxation process which couples to the acoustic waves. These equations suggest that the anomalous changes in the real and imaginary part of the elastic constant, which is related to the Brillouin shift and the FWHM, respectively, are proportional to the squared polarizations of PNRs. As Figure 2 shows, the anomalous changes in *ν*_B_ and Γ_B_ are largest along the <100> direction and smallest along the <111> direction. Thus, the polarization fluctuations of PNRs are anisotropic, showing largest polarization fluctuations along the <100> direction. Similar results were obtained in the case of PMN-PT, where both Re[Δ*C*] and Im[Δ*C*] increase with increasing PT content on the rhombohedral side [33]. It is well known that the magnitude of the local polarization of PNRs increases upon approaching the MPB in the PMN-PT system. Regarding the above two equations, it should be noted that the electrostrictive coefficient *γ* may depend on crystallographic direction, and thus the anisotropy of the electrostrictive coefficient may also have an effect on the anomalous changes of the elastic constant.

**Figure 6:**
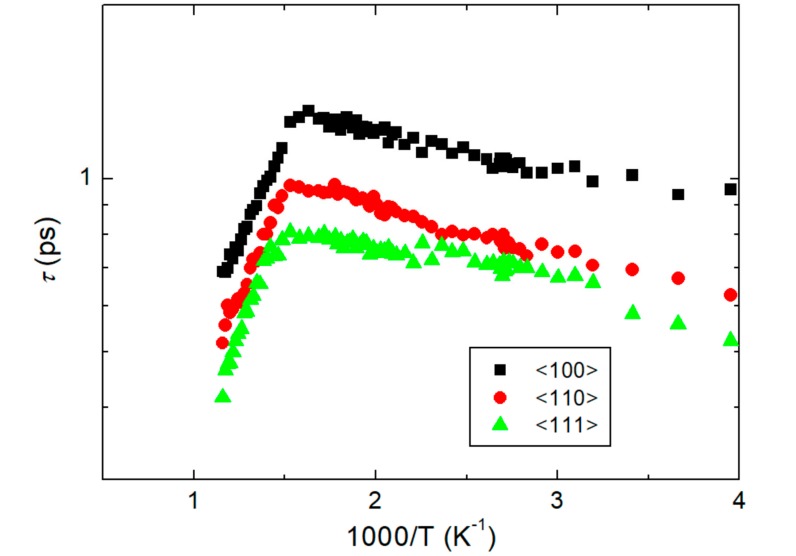
Relaxation time estimated by using Equation (3) for the three crystallographic axes.

The relaxation time of the polarization fluctuations can be derived from the measured acoustic behaviors by using Equation (3) [37],

(3) $$\tau =\frac{\Gamma_{B}-\Gamma_{\infty }}{2\pi \left[\nu_{\infty }^{2}-\nu_{B}^{2}\right]},$$

where $\nu_{\infty }^{}$ is the Brillouin shift of the unrelaxed system at zero order parameter. In this equation, the ($\Gamma_{B}-\Gamma_{\infty }$) should represent the excess damping caused by polarization fluctuations, that is, the measured half width minus the constant value of the flat region at high temperatures. Equation (3) is valid only for the relaxation process of a single relaxation time, which should be valid at high temperatures of NBT-*x*BT. Figure 6 shows the temperature dependence of the relaxation time in the Arrhenius plot. The *τ* increases upon cooling from the high-temperature side toward the dielectric maximum temperature. The temperature dependences of the relaxation time in the paraelectric phase for the three crystallographic axes are similar with slight differences. The relaxation time along the <100> direction is the largest while that along the <111> direction is smallest. This difference is associated with the suggestion that the magnitude of the polarization fluctuation is largest along the <100> direction as the Re[Δ*C*] and Im[Δ*C*] exhibit the most substantial changes. The temperature dependence of *τ* cannot be described in terms of the critical slowing-down behavior, which reflects the order–disorder component of typical ferroelectrics, such as BaTiO_3_ [38]. This again indicates that the dynamics of polar clusters of unpoled NBT-*x*BT near MPB exhibit typical relaxor properties instead of critical divergent behaviors.

Electric-field-induced structural changes in NBT-5%BT show drastic changes in the elastic properties at ~110 °C when the poling field is larger than 1.4 kV/mm. The Brillouin shift of the LA mode drops suddenly while its half width exhibits a sharp damping peak at this temperature. Jo et al. suggested that the depoling process occurs via a two-stage process, that is, the ferroelectric domains first lose their ferroelectric and ferroelastic texture and then the detextured ferroelectric domains are transformed into nanoscale polar regions at higher temperature [18]. Figure 5 shows that the acoustic properties of prepoled samples become exactly the same as those of unpoled NBT-*x*BT at ~110 °C. This indicates that the characteristic temperature of ~110 °C corresponds to the ferroelectric-to-relaxor transition, and detexturization of ferroelectric domains at lower temperature does not have any substantial effect on acoustic properties. This result is in line with the results of PMN-PT [33]. PMN-31%PT near MPB shows a sharp damping peak at the cubic-tetragonal phase transition. It was also attributed to the percolation transition representing the transformation of mesoscopic/macroscopic ferroelectric domains into PNRs.

## 4. Materials and Methods

### 4.1. Materials

NBT-*x*BT single crystals were grown by carefully controlled top-seeded solution growth method. The details of the crystal growth can be found elsewhere [19,20]. The grown single crystals were cut into a (100), (110), or (111) plate. The typical dimensions of the plates were 4 × 4 × 1 mm^3^. The two largest surfaces were polished to optical quality. In most cases, poling was not carried out for the crystals before the Brillouin scattering measurement. When poling was necessary, silver paste was used to form electrodes on the crystal surfaces, which was fired at 200 °C for 30 min. Gold lines were connected to the electrodes. The crystal was inserted in silicone oil and an electric field was applied to the crystal for 30 min by using a high voltage power supply (Bi-polar HV Power Supply, FTLab, Anyang, Korea).

### 4.2. Methods

The crystal plate was inserted in a compact temperature controller (THMSE600 or TS1500, Linkam, Tadworth, UK) which was put under a modified microscope (BX-41, Olympus, Tokyo, Japan) for backscattering measurements. The Brillouin scattering spectrum was measured by using a conventional tandem six-pass Fabry-Perot interferometer (TFP-1 or TFP-2, JRS Co., Zürich, Switzerland) and a diode-pumped solid state laser (Excelsior 532-300, Spectra Physics, Santa Clara, CA, USA). The wavelength of the laser was 532 nm, operated at the power of tens of mW. The unpoled NBT-5%BT is pseudo-cubic without any measurable birefringence. The polarization direction of the incident laser beam was along the pseudo-cubic [100] direction. The free spectral range of the interferometer was set to 75 GHz for probing the acoustic modes. A conventional photon-counting system combined with a multichannel analyzer (1024 channels) was used to detect and average the signal. All the Brillouin spectra were measured with increasing temperature. The details of the experimental setup can be found elsewhere [39,40].

## 5. Conclusions

The acoustic properties of unpoled and prepoled (Na_1/2_Bi_1/2_)TiO_3_-*x*BaTiO_3_ (NBT-*x*BT) single crystals near the morphotropic phase boundary were investigated over a wide temperature range using Brillouin spectroscopy. The Brillouin mode frequency and the damping factor of the longitudinal acoustic mode of NBT-*x*BT with *x* = 0.05 showed broad minimum and maximum, respectively, which denotes that the unpoled NBT-*x*BT near the MPB exhibit typical relaxor behaviors. The degree of mode softening and the damping height were most substantial along the <100> direction, which indicates that the magnitude of the polarization fluctuations was largest along this direction. A single relaxation process for the polar nanoregions was assumed in the high-temperature ergodic relaxor phase, based on which the temperature dependence of the relaxation time was obtained. The relaxation time increased gradually on approaching the dielectric maximum temperature upon cooling from the high-temperature side. NBT-*x*BT prepoled along the <100> direction at room temperature induced discontinuous changes in the elastic properties at ~110 °C once the poling field became larger than 1.4 kV/mm, corresponding to the depoling process from macroscopic/mesoscopic ferroelectric order to ergodic relaxor state upon heating. Both ferroelectric and relaxor states coexisted at low temperatures when NBT-*x*BT was prepoled at the intermediate poling field of 1.4 kV/mm.
